# Case report: Brucellosis with rare multiple pulmonary nodules in a depressed patient

**DOI:** 10.3389/fmed.2022.1111830

**Published:** 2023-01-17

**Authors:** Mingjing Zhou, Ke Wang, Haoyuan Liu, Ran Ran, Xuan Wang, Yuqian Yang, Qunying Han, Yi Zhou, Xiaojing Liu

**Affiliations:** The First Affiliated Hospital of Xi’an Jiaotong University, Xi’an, China

**Keywords:** brucellosis, pulmonary nodules, depression, developing countries, case report

## Abstract

**Background:**

Brucellosis is a zoonotic disease that threatens public health and creates an economic burden. Unfortunately, it is often overlooked in developing countries, with misdiagnosis causing negative impacts on those with low income. Although the symptoms of brucellosis are commonly reported as fever and fatigue, rare pulmonary, and psychiatric involvements should also be considered. We present the first brucellosis patient in China with multiple pulmonary nodules and depression. Furthermore, this report highlights the importance of collecting patient history in epidemic areas of brucellosis.

**Case presentation:**

We report the case of a 40-year-old woman with intermittent fever for 2 months and gradually accompanied by chills, dry cough, arthralgia, and fatigue. The patient was also diagnosed with depression after fever. She received symptomatic treatment at a regional hospital; however, there was no significant symptom relief. She suddenly developed hemoptysis 1 day prior to arrival at our hospital, where we discovered that her liver, spleen, neck, and axillary lymph nodes were enlarged, and there were multiple nodules in both lungs. The patient was eventually diagnosed with brucellosis after the serum agglutination test and received antibiotic therapy, which provided symptom relief.

**Conclusion:**

This report describes a case of brucellosis with uncommon multipulmonary nodules and depression in China. This study has widened the evidence of respiratory involvement due to brucellosis. Second, it demonstrates the importance of collecting a comprehensive medical history, especially in epidemic areas. In conclusion, for febrile patients with pulmonary nodules and depression, especially in endemic areas, brucellosis should be considered.

## 1. Introduction

Brucellosis is a common universal zoonosis caused by *Brucella*, a gram-negative bacterium that affects sheep, cattle, and pigs, and is transmitted to humans through the skin mucosa, respiratory tract, and digestive tract (1). Patients with brucellosis generally present with fever, fatigue, chills, and swelling. Occasionally, the bacterium can invade the nerve systems, which may cause various neuropsychiatric manifestations (2, 3). However, pulmonary involvement is a rare complication of brucellosis (4, 5).

More than 500,000 new cases are reported annually, especially in developing countries (6). Similar to China, the prevalence of brucellosis tends to be higher in northern cities and was closely associated with livestock during 2004–2018 (7). However, owing to lack of financial and medical resources, brucellosis remains neglected in developing countries. Misdiagnosis of brucellosis will significantly impose heavy economic burden on public health systems (1, 8, 9). In this report, we describe a Chinese depressed patient with rare multi-pulmonary nodule brucellosis and present the medical history, symptoms, signs, and results of a series of examinations. The patient was diagnosed with brucellosis and received effective antibiotic therapy. This report highlights the importance of clinical-epidemiological suspicion as well as early diagnosis of brucellosis.

## 2. Case description

On July 10, 2022, a 40-year-old woman was admitted to the first affiliated hospital of Xi’an Jiaotong University due to intermittent fever for 2 months. Two months prior, her temperature fluctuated between 37.4 and 38.4°C in the afternoons, and chills and a dry cough accompanied this symptom. She had previously undergone examination and symptomatic treatment at a regional hospital. However, the treatment was ineffective. Furthermore, arthralgia and fatigue gradually appeared, with no chest pain and tightness, or rash. A month and a half prior, she was diagnosed with depression and treated with escitalopram oxalate, and magnesium valproate sustained-release tablets at a hospital. The symptoms slightly improved after treatment. Half a month prior, a B-ultrasonic examination showed lymphadenopathy throughout her body. Therefore, lymphoma was suspected but she did not get any treatment. One day prior to her arrival at our hospital, the patient suddenly developed hemoptysis of approximately 2 ml, which occurred four times for no reason. Past medical history revealed that she was diagnosed with chronic hepatitis B (CHB) in 2014 and developed cirrhosis a year later. Her CHB was well-controlled by a long-term oral antiviral treatment with Entecavir. In addition, she had lost 5 kg over the past 3 months.

On admission, her temperature was 37°C, respiratory rate was 19 breathe⋅min^–1^, heart rate was 70 beat⋅min^–1^, and blood pressure was 103/72 mmHg. The liver, spleen, lymph nodes of the neck, and axillary lymph nodes were enlarged with moderate activity and no tenderness. Rough breath sounds were detected in both lungs with dry and wet rales. All other clinical examination results were negative.

Laboratory examination revealed inflammation and liver dysfunction: red cell count, 4.27 × 10^12^/L (4–4.5 × 10^12^/L); white cell count, 4.87 × 10^9^/L (5–12 × 10^9^/L); platelet count 89 × 10^9^/L (125–350 × 10^9^/L), hypersensitive C-reactive protein 9.81 mg/L (0–3 mg/L), direct bilirubin 6.2 μmol/L (0–3.4 μmol/L), aspartate aminotransferase, 54 U/L (13–45 U/L); alanine aminotransferase, 20 U/L (7–40 U/L); alkaline phosphatase, 164 U/L (35–100 U/L); γ-glutamyl transpeptidase, 82 U/L (7–45 U/L), albumin 33.8 g/L (40–55 g/L).

**FIGURE 1:**
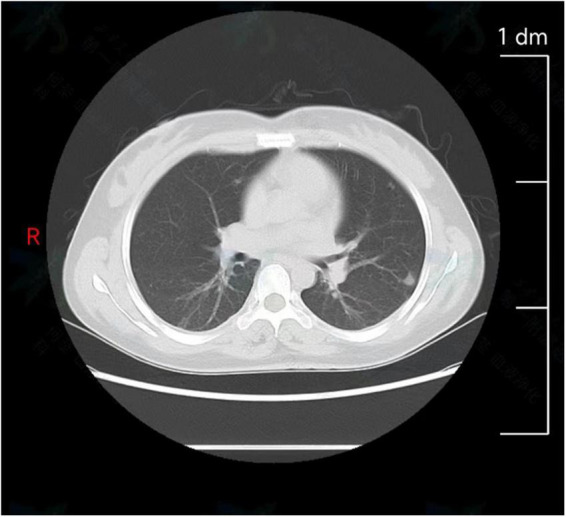
Chest computed tomography (CT) revealed multiple pulmonary nodules in both lungs.

B-scan ultrasonography revealed bilateral cervical and axillary lymphadenopathies. Chest computed tomography (CT) revealed multiple pulmonary nodules in both lungs, with a small amount of pleural effusion (Figure 1) and enlarged lymph nodes in the bilateral axilla. To effectively confirm the nature of the lesion, we performed needle biopsies of pulmonary nodules and lymph nodes. The results implied an infectious disease, while the detection of T cells in tuberculosis infection was negative. Therefore, suspicions of tumors and tuberculosis were excluded.

Interestingly, we noticed that the patient came from Ningxia province, which is famous for animal husbandry. The patient reluctantly informed us that she raised cattle and had come into contact with neighboring sheep without vaccination. Consequently, *Brucella* infection was suspected. On the fourth day of admission, the serum agglutination test (SAT) result was 1:800, and the rose-bengal plate agglutination test (RBPT) result was positive. Furthermore, the blood culture for *Brucella melitensis* was positive on the tenth day after admission. Characteristic rod-shaped gram-negative bacteria could be observed under a microscope. Subsequently, the patient was definitively diagnosed with brucellosis.

**FIGURE 2:**
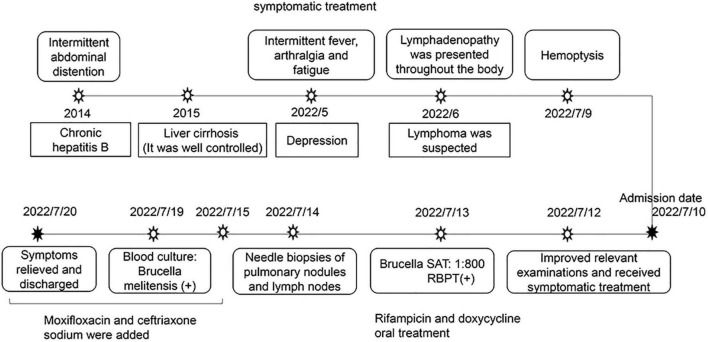
The entire process of diagnosis, treatment, and outcome.

Following the brucellosis diagnosis, she received antibiotic therapy with rifampicin (600 mg/dose, once a day) and doxycycline (100 mg/dose, twice a day) for 3 months from the fourth day of the course. Furthermore, due to the poor medical conditions of the patient’s residence and excessive complications, including multipulmonary nodules, arthralgia, hepatosplenomegaly, and lymphadenopathy, moxifloxacin and ceftriaxone sodium were added from the sixth day of the course to prevent the possibility of developing drug-resistant brucellosis after discharge. During treatment, the patient and her family were highly cooperative. At discharge, fever, cough, arthralgia, depression and fatigue were relieved. After 2 months of follow-up, the fever and cough were gone, as was fatigue and arthralgia. In addition, the number of multiple nodules in both lungs was reduced. At the same time, liver function test results also indicated that the patient was recovering well. After 3 months of follow-up, her weight had increased and her depression symptoms were alleviated. The entire process of diagnosis, treatment, and outcomes is shown in Figure 2.

## 3. Discussion and conclusion

Brucellosis is a zoonosis with more than half a million newly reported cases worldwide each year, posing a threat to public health and increasing economic burdens (1, 9). In particular, owing to a lack of resources, this remains a neglected disease in developing countries. In China, brucellosis cases continue to increase and have appeared in all provinces (10, 11). Severe epidemic areas include Jilin, Xinjiang, Qinghai, Ningxia, and Henan, and April to June is considered to be peak epidemic periods (11). In this report, we present the first publicly reported brucellosis patient in China with multiple pulmonary nodules and depression. More importantly, due to signs of multipulmonary nodules and lymphadenopathy, the patient was misdiagnosed with a tumor, which increased her economic and psychological burden.

**FIGURE 3:**
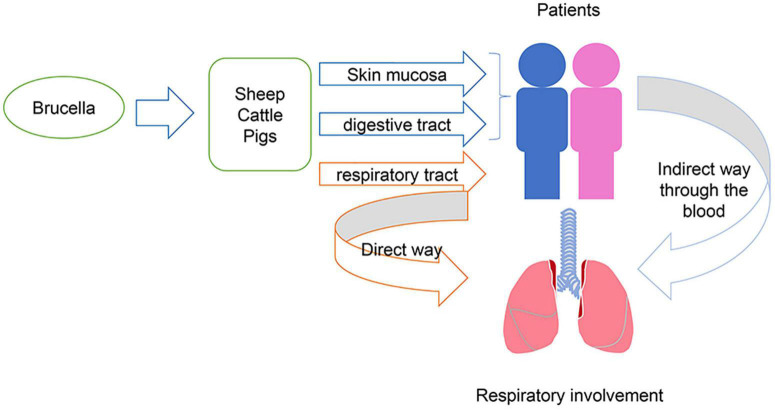
Mechanism of respiratory involvement by *Brucella*.

In general, brucellosis causes fever, fatigue, and chills. However, an expanded understanding of the disease has led to reporting of an increasing number of complications. These include respiratory (4), ocular (12), nerve (13), and reproductive systems (14). Regarding respiratory involvement, brucellosis can present with bronchitis, pleural effusion, and pulmonary nodules (15–17). These symptoms may be directly caused by the transmission of bacteria through the respiratory tract to the respiratory system, or they can be caused by the transmission of bacteria through the blood after infecting the human body in other ways (Figure 3). Respiratory involvement due to brucellosis in China has rarely been reported. A previous study found that the percentage of respiratory involvement in Chinese brucellosis patients was only 13% (18). This is insufficient to provide comprehensive evidence for an accurate diagnosis of brucellosis in the future.

Pulmonary nodules are mostly benign and can be caused by many factors, including cancer, infection, and inflammation (19). Pulmonary metastatic carcinoma is the most common cause of cancer and is usually accompanied by cachexia. Etiological and immunological tests often provide evidence of infection and inflammation. To make a clear diagnosis, we performed needle biopsies of pulmonary nodules under CT guidance, and the results suggested an infectious disease. Blood cultures for *Brucella*, SAT, and RBPT were also positive. By combining the patient’s symptoms, signs, and epidemic history, a diagnosis of brucellosis was confirmed.

Interestingly, the patient was diagnosed with depression after presenting with fever, arthralgia, and fatigue. Several studies have reported that depression was one of the rare symptoms of brucellosis infection (20, 21). In this case, the patient’s emotional changes such as mood disorder, feelings of sadness and loss of interest were slightly improved after anti-depression therapy while were alleviated by antibiotic therapy. Considering her epidemiological history and psychiatric manifestations after intermittent fever, we presumed that her depression might be caused by brucellosis. Her head MRI scans and neurological signs were both negative. Unfortunately, further examinations to evaluate her neuropsychiatric manifestations were not carried out, which is a major limitation in our study.

Antimicrobial therapy is usually chosen for the treatment of brucellosis. Doxycycline combined with rifampicin is considered the most common combination choice in the treatment of pulmonary brucellosis (22–24). In cases with complications, treatment should be appropriately extended. In this report, considering the lung involvement and multiple lymphadenopathies throughout the body, the patient was treated with quadruple antibacterial treatment, including rifampicin, doxycycline, ceftriaxone sodium, and moxifloxacin. Due to lack of a definite diagnosis of neurobrucellosis, we decided to give this patient the 3 months standard treatment regimen. Her symptoms were alleviated a lot and did not recur after 3 months antibiotic therapy.

In conclusion, we reported a case of brucellosis with multipulmonary nodules in a depressed patient. Our report adds to the literature on the complications of brucellosis, which is often neglected in developing countries. During the process of diagnosis, we demonstrated the importance of obtaining a complete personal history of patients from disease-endemic areas. In addition, we suggest that examinations of *Brucella* should be carried out among patients with fever, pulmonary involvement and depression in endemic areas.

## Data availability statement

The original contributions presented in this study are included in this article/supplementary material, further inquiries can be directed to the corresponding authors.

## Ethics statement

The studies involving human participants were reviewed and approved by the Ethical Review Committee of Xi’an Jiaotong University. The patients/participants provided their written informed consent to participate in this study. Written informed consent was obtained from the individual(s) for the publication of any potentially identifiable images or data included in this article.

## Author contributions

MZ, QH, YZ, and XL designed the study. KW and HL collected the clinical data. RR, XW, and YY analyzed the data and provided the materials. MZ wrote the first draft of the manuscript. All authors participated in the diagnosis and treatment of the patient, read, and approved the final manuscript.
